# Schizophrenia and substance use comorbidity: a genome-wide perspective

**DOI:** 10.1186/s13073-017-0423-3

**Published:** 2017-03-21

**Authors:** Renato Polimanti, Arpana Agrawal, Joel Gelernter

**Affiliations:** 1 Department of Psychiatry, Yale School of Medicine and VA CT Healthcare Center, West Haven, CT USA; 2 Department of Psychiatry, Washington University School of Medicine, St. Louis, MO USA; 3 Department of Genetics and Department of Neuroscience, Yale School of Medicine, New Haven, CT USA

## Abstract

Dual diagnosis with substance use disorders (SUDs) consistently contributes to the premature mortality and increased disability observed in schizophrenia. Large genome-wide association studies are providing the information needed to investigate the genetic architecture of psychiatric disorders. Here, we discuss recent genetic investigations of dual diagnosis (i.e., schizophrenia plus a SUD) and how these findings can inform public health messages.

## Theories of dual diagnosis

The comorbidity between a mental illness and a substance use disorder (SUD; i.e., a condition in which the use of one or more substances leads to a clinically relevant impairment or to distress) is usually defined as a dual diagnosis. This is frequent, dangerous and problematic when the psychiatric illness is schizophrenia (in this article, we will refer to comorbidity specifically of schizophrenia and a SUD as “dual diagnosis”). Individuals with schizophrenia are more likely than those without the disorder to smoke, heavily use alcohol, heavily use cannabis, and use recreational drugs; these factors contribute to the premature mortality and increased disability observed in patients with schizophrenia [1]. Accordingly, understanding the pathogenesis of dual diagnosis as an entity has the potential to improve clinical outcomes. There are four major theories to explain the prevalence of dual diagnosis. First, the “self-medication” hypothesis suggests that individuals with schizophrenia develop a SUD because they feel it improves some of their symptoms. A second possibility is that schizophrenia and SUDs are influenced by a common pathophysiology (i.e., shared genetic mechanisms). Third, in the diathesis-stress model, genetic predisposition interacts with chronic substance use, and this interaction results in the onset of schizophrenia. A final theory is that impaired social and occupational function due to schizophrenia together with exposure to poor social environments coincide with an increased risk of substance use. Numerous approaches have been used to investigate these hypotheses, but substantive progress in understanding the underlying genetic architecture has been made only recently. Here, we describe the results from genome-wide association studies (GWAS) and discuss how this information can be used to test the above-mentioned hypotheses regarding the pathogenesis of dual diagnosis in schizophrenia.

## Genetic overlap between schizophrenia and SUDs

Early family studies provided mixed evidence regarding the co-aggregation of SUDs in the relatives of schizophrenic probands. GWAS are now contributing greatly to our understanding of the genetic predisposition to complex traits. GWAS test the association of common variation across the entire human genome, and can therefore generate new hypotheses or provide unbiased information to support or refute old ones. For instance, GWAS of substance dependencies showed evidence of local genetic overlap and identified risk genes that had previously been associated with schizophrenia risk [2]. In 2014, the Schizophrenia Working Group of the Psychiatric Genomics Consortium published a meta-analysis of GWAS that included up to 36,989 cases and 113,075 controls, and identified 108 novel genetic loci associated with the risk of schizophrenia [3]. Data from this study boosted research in schizophrenia genetics more generally, providing investigators with the resources needed to study pleiotropy and to conduct post-GWAS analyses. Summary statistics from this GWAS were used to perform polygenic risk score (PRS) analyses to investigate the global genetic overlap between schizophrenia and many other traits, including SUDs. For instance, addressing the uncertain association between cannabis use and schizophrenia, the overall genetic correlation with summary statistics from a recent large meta-analysis of cannabis use (*r*_g_) is 0.22 [4, 5], which supports the existence of genetic mechanisms that are shared between these two traits. A PRS study from deCODE Genetics conducted on 144,609 Icelandic subjects tested the association of schizophrenia PRSs with several SUD-related traits; the authors observed significant associations for disorders of alcohol, amphetamine, cocaine, cannabis, opioid and sedative use, as well as an early onset of addiction, smoking initiation, Fagerström Test for Nicotine Dependence, number of cigarettes smoked per day, and the number of hospital admissions for in-patient addiction treatment (Nagelkerke’s *R* ^2^ 0.08 to 0.89%) [6]. Collectively, these results indicate that shared covariance from *common* genetic variation is implicated in the comorbidity of schizophrenia and SUDs. However, on the basis of these genetic correlations alone, no conclusions can be drawn regarding the mechanisms that link these complex traits.

## Causal relationships

Mendelian randomization (MR) methods offer one approach to investigating causality in associations between schizophrenia and SUDs. A form of instrumental variable analysis, MR refers to the random assignment of an individual's genotype at conception. If sets of genetic variants can robustly predict an exposure of interest (e.g., drawn from a well-powered GWAS), they can be used as unconfounded proxies of the exposure (i.e., instruments) and tested with respect to a specific outcome, assuming that possible pleiotropy is accounted for.

Two studies have used MR methods to investigate causality in the association between cannabis use and schizophrenia [7, 8]. Vaucher et al. reported that the top ten loci from a GWAS of cannabis use were associated with an increased risk of schizophrenia, when tobacco exposure and pleiotropy were taken into account [7]. Gage et al. found similar results and reported a modest effect (odds ratio (OR) <1.07) of genetic liability to cannabis use on the risk of schizophrenia [8]. However, they also reported a stronger “causal” effect of genetic liability to schizophrenia on cannabis use (OR = 1.10). This asymmetry in the association might be attributable to multiple factors. First, cannabis use may represent an imprecise and relatively heterogeneous phenotype. For instance, one of the largest GWAS of cannabis dependence symptoms failed to find genome-wide evidence for pleiotropy with schizophrenia [2]. Second, the reduced sample size of the cannabis dependence GWAS with respect to the schizophrenia GWAS (32,330 versus 150,064 individuals, respectively [3, 4]) might have attenuated the predictive utility of the cannabis instrument; that is, the directional predictive power was asymmetrical. Third, the association might reflect evidence for reverse causation (i.e., suggesting that genetic liability to schizophrenia results in the onset of the disorder, which further contributes to an escalation of cannabis use). Alternatively, consistent with prior PRS studies [5], these limited-power MR analyses may not have fully eliminated the role of a shared genetic architecture underlying the comorbidity between cannabis use (or other SUDs) and schizophrenia.

## Gene–environment interplay

An alternative to both causation and pleiotropy is that early-onset and heavy cannabis use might trigger an underlying genetic liability to schizophrenia. Using PRSs from the large Psychiatric Genomics Consortium schizophrenia GWAS, a 2015 study found that an increasing genetic liability to schizophrenia in males was associated with decreasing cortical thickness, which represents a possible endophenotype (i.e., a trait that should be heritable, should co-segregate with a psychiatric disorder yet be present even when the disease is not, and should be found in non-affected family members at a higher rate than in the population), but only as a function of increasing cannabis use during adolescence [9]. Despite the appeal of such gene-by-environment models, the power to detect interactions is reduced compared with the power of GWAS to detect genetic associations, especially given the small effect sizes attributable to individual loci or PRSs.

## Future investigations

If schizophrenia and SUDs share genetic underpinnings, then this finding strongly challenges the rigid diagnostic boundaries that separate these psychiatric disorders and might have clinical implications. However, genome-wide meta-analyses of SUDs on the same scale as those of schizophrenia are lacking. Similarly to their schizophrenia counterpart, the newly established SUD Working Group of the Psychiatric Genomics Consortium aims to analyze the largest compilation of GWAS data for a variety of SUDs [10]. We are highly confident that discoveries from these collaborative groups will deliver the genetic instruments necessary to investigate the mechanisms responsible for dual diagnosis in schizophrenia with better power than is attainable today. This expected genetic information will provide unbiased evidence that will be useful when addressing relevant public health issues, such as the consequences of cannabis use in the general population.

## Abbreviations

GWAS: genome-wide association studies
MR: mendelian randomization
OR: odds ratio
PRS: polygenic risk score
SUD: substance use disorder
